# Clinical outcomes of traumatic globe rupture in corneal graft patients

**DOI:** 10.1007/s10792-024-03008-w

**Published:** 2024-02-29

**Authors:** Francisco Figueiredo, Jas Sandhu, Michael Shaw

**Affiliations:** 1 Department of Ophthalmology, Royal Victoria Infirmary, Newcastle upon Tyne Hospitals NHS Foundation Trust, and Bioscience Institute, Faculty of Medical Sciences, Newcastle University, Newcastle upon Tyne, UK; 2 Department of Ophthalmology, Royal Victoria Infirmary, Newcastle upon Tyne Hospitals NHS Foundation Trust, Newcastle upon Tyne, UK; 3 Department of Ophthalmology, Gozo General Hospital, Gozo, Malta

**Keywords:** Trauma, Globe rupture, Keratoplasty, Wound dehiscence, Clinical outcomes

## Abstract

**Purpose:**

This study reports the mechanisms, complications and graft survival following sight-threatening traumatic globe rupture in patients having previously undergone corneal transplantation in the same eye.

**Methods:**

A retrospective, observational, single-center consecutive cohort study at the Royal Victoria Infirmary, Newcastle upon Tyne, UK over a 20-year period. Medical records and Newcastle Corneal Transplantation Service electronic database (eNCTS) review was undertaken of all consecutive patients who underwent corneal transplantation with a history of traumatic globe rupture.

Main outcome measures include mechanism of injury, final best-corrected distance visual acuity (BCDVA), graft survival and complications.

**Results:**

A total of 921 keratoplasties were undertaken between 1997 and 2017 with 24 (3.0%) patients identified with a history of traumatic globe rupture.

A bimodal relationship of age and mechanism of trauma was observed. The mean age (SD) of individuals reporting cause as a fall was 71.5 (14.8) years, and 45.3 (20.8) years (*P* < 0.05) amongst individuals reporting accidental trauma or assault.

The pre- and post-trauma mean (SD) LogMAR BCDVA was 0.6 (0.9) and 1.7 (1.0), respectively (*P* = 0.001). The overall graft-failure rate was 60.9% (11 grafts) during a mean (SD) follow-up period of 3.5 (4.1) years. Globe rupture with lens damage was associated with poorer final BCDVA (*P* < 0.05).

**Conclusions:**

This study represents the first published series from England for this type of patient cohort. Overall visual outcomes were poor with a bimodal relationship of age and mechanism of trauma. Worse prognostic factors included lens and posterior segment complications. Re-grafting in these select group of patients may prove valuable.

## Introduction

Corneal transplantation (keratoplasty) is the most common and successful form of transplantation in humans [1]. As a result of on-going improvements and developments in surgical techniques and in the post-operative management, keratoplasty surgery is considered to be a relatively safe and a rather successful transplantation procedure using different surgical techniques for a broad range of indications and offers good visual outcomes [2].

Over the last few years, there has been an increasing trend towards undertaking lamellar keratoplasties (e.g., deep anterior lamellar and endothelial keratoplasties) as a result of the associated potential benefits, amongst these is the post-operative maintenance of globe integrity. Full thickness keratoplasties still remain widely performed when indicated [3], however are associated with impaired strength and structural globe stability [4]. Consequently, potentially even minor trauma may result in globe rupture and severe intraocular injury [5]. In these patients, the visual outcomes and graft survival reports are seldom good [6].

The purpose of this study is to report the incidence, mechanisms, patients’ characteristics, risk factors, complications, graft survival and visual outcomes following severe trauma with globe rupture in consecutive patients having previously undergone keratoplasty in the same eye, i.e., penetrating (PK), deep anterior lamellar (DALK) and Descemet’s stripping automated endothelial (DSAEK) keratoplasties at a single tertiary referral centre in the UK over a 20 year period (1997–2017).

## Materials and methods

We present a retrospective observational single-center cohort study undertaken in a tertiary corneal transplantation service in the UK between January 1997 and December 2017 of all consecutive patients having had an episode of traumatic globe rupture following corneal transplantation in the same eye. All keratoplasties were undertaken by the senior author (FCF), or under his direct supervision using similar techniques for all patients as described by Figueiredo et al. [7] In brief, penetrating keratoplasties were performed using 10/0 interrupted nylon sutures (12 sutures; Ethicon, UK) combined with 11/0 nylon continuous suture (12 bites; Ethicon, UK) and the donor graft button was oversized by 0.25 mm in diameter for all patients. DALKs were performed using the previously reported Melles’ Technique with minor modifications [8] and DSAEKs were performed using the technique reported by Müller et al. [9].

The data regarding all the patients who underwent consecutive corneal grafts were collected prospectively using a local electronic database [Newcastle Corneal Transplantation Service electronic database (eNCTS)] and additional trauma related data were collected retrospectively from medical records. The tenets of the Declaration of Helsinki were followed in this anonymized study, and approved by the Clinical Governance and Audit Department of the Newcastle upon Tyne Hospitals NHS Foundation Trust for a systematic and continuous data collection and audit of all corneal transplantation surgery outcomes. All patients were consented prior to their corneal transplantation for data collection and analysis accordingly. The study included all consecutive patients of all ages who sustained a severe blunt injury with globe rupture following corneal grafting in the same eye with a mean (SD) follow-up of 3.5 (4.1) years, range 3 months and 16 years.

Patients’ medical records and an electronic database were reviewed and outcome measures recorded including patient demographics, indication for grafting, keratoplasty type, lens status, latency period from surgery to trauma, mechanism of trauma, final best corrected distance visual acuity (BCDVA) and graft status outcomes and associated complications. Snellen chart BCDVA values were converted into LogMAR scale equivalent for analysis purposes.

Globe rupture wounds were repaired as an emergency under GA using 10/0 interrupted nylon sutures (Ethicon, UK) and further surgery was performed as judged necessary.

### Statistical analysis

All data analyses were performed using statistical analyses with Microsoft Excel (Microsoft Corporation, Washington, USA). Fisher’s exact test with a two-tailed *P* value was used to analyse categorical data with small sample sizes. The Mann–Whitney U-Test was used for statistical non-parametric testing. Differences were considered statistically significant when the* P* value was < 0.05.

## Results

A total of 921 keratoplasties had been undertaken over the 20 years study period, including *n* = 804 (87.3%) PKs, *n* = 27 (2.9%) DALKs and *n* = 90 (9.8%) DSAEKs. A total of 24 (3.0%) patients were identified with a previous history of traumatic globe rupture, of these 3 patients had incomplete data, and were excluded from the analysis. This brings it to a total of 21 patients with 22 separate episodes of eye trauma. One patient suffered two separate episodes of ruptured globe, 10 years apart in the same eye, and required two separate PKs as the patient needed re-grafting 8 years from the first trauma episode. All episodes of globe rupture occurred in patients having previously undergone PK surgery and no cases of previous DALK or DSAEK procedures. A detailed account of this series is reported in Tables 1 and 2.

**Table 1:** Corneal graft demographics, occupational status and ocular co-morbidities

Demographics	*n* (%)
Male gender	12 (57.1)
Mean age at PK, years (SD)	53.4 (20.7)
Mean age at trauma, years (SD)	56.9 (22.0)
Mean duration of follow-up post injury, years (SD)	3.5 (4.1)
Occupational status	
Employed	8 (38.1)
Retired	7 (33.3)
Unemployed	2 (9.5)
Child	1 (4.8)
Unknown	3 (14.3)
Concurrent ocular co-morbidities	
Glaucoma	4 (19.0)
Strabismus	3 (14.3)
Cataract	2 (9.5)
Uveitis	1 (4.8)
Severe PDR*	1 (4.8)
Dry AMD^Δ^	1 (4.8)
None	16 (76.2)

*Proliferative diabetic retinopathy

^Δ^Age-related macular degeneration

**Table 2:** Original indication for corneal graft surgery and previous number of corneal graft surgeries

Indication for keratoplasty	*n* (%)
Fuchs’ endothelial dystrophy	4 (19.0)
Keratoconus	3 (14.3)
Traumatic corneal scarring	2 (9.5)
Pseudophakic bullous keratopathy	2 (9.5)
Corneal scar due to LSC^∞^ failure from chemical injury (post autologous LSC Transplantation)	2 (9.5)
Corneal melt/perforation due to chemical injury (therapeutic PK)	2 (9.5)
Aphakic bullous keratopathy	1 (4.8)
Iridocorneal endothelial syndrome	1 (4.8)
Pellucid marginal degeneration	1 (4.8)
Corneal scar due infectious keratitis	1(4.8)
Lattice dystrophy type 1	1(4.8)
Peter’s Anomaly	1(4.8)
Previous number of graft surgeries	
1	17 (81.0)
2 or more	4 (19.0)

^∞^Limbal stem cell

### Eye characteristics prior to trauma

Time between keratoplasty and trauma (latency), keratoplasty status, graft suture status and lens status, but also mechanism of trauma and location of the trauma episodes are all reported in Tables 3 and 4, respectively. No patients suffered bilateral trauma. Keratoplasty failure occurred pre-trauma in three patients, two secondary to acquired infection and one due to a recurrence of lattice dystrophy. The circumstances of the trauma in the majority of episodes were accidental, with 14 (63.6%) of the 22 occurring in the home setting. The most common cause of injury was as a result of falling, occurring in 9 patients (40.9%). The mean age of those falling compared to other modes of injury was significantly higher at 71.5 years (SD 14.8) and 45.3 years (SD 20.8), respectively, *P* < 0.05.

**Table 3:** Trauma latency, keratoplasty status, suture status, lens status

Latency	
Time from keratoplasty to trauma (years)	*n* (%)
< 0.5	4 (18.2)
0.5 – 1	3 (13.6)
1 – 2	2 (9.1)
2 – 5	8 (36.4)
5 – 10	5 (22.7)
Mean time from keratoplasty to trauma, years (SD)	7.7 (2.5)
Keratoplasty status before trauma	
Clear	18 (81.8)
Failed	3 (13.7)
Not known^Δ^	1 (4.5)
Type of suture and status at time of trauma	
Continuous + interrupted sutures in place	11 (50.0)
Continuous + part interrupted in place	4 (18.2)
Interrupted only in place	2 (9.1)
Part interrupted only still in place	1 (4.5)
Not known	4 (18.2)^Δ^
Lens Status	
PC IOL	10 (45.6)
Phakia	7 (31.8)
Aphakia	3 (13.6)
AC IOL	1 (4.5)
Not known^Δ^	1 (4.5)

^Δ^Data unavailable

**Table 4:** Mechanism and location of injury episodes

Mechanism	*n *(%)
Falls	9 (40.9)
DIY^α^ *	4 (18.2)
Accidentally poked in the eye	7 (31.8)
Assault	1 (4.5)
Not known^Δ^	1 (4.5)
Location	
Home	14 (63.6)
Work	1 (4.5)
Not known^Δ^	7 (31.8)

*All injuries were blunt except for one penetrating injury with a screwdriver in the DIY category

^α^DIY: Do it yourself

^Δ^Data unavailable

### Trauma associated ocular outcomes

#### Visual acuity

**Fig. 1:**
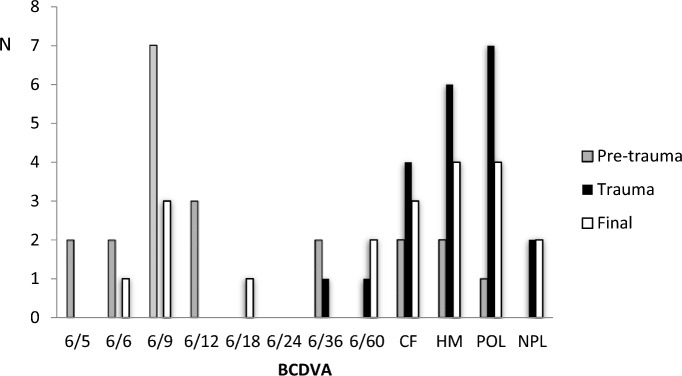
BCDVA pre-trauma, at the time of trauma and final in the grafted eye

The mean BCDVA (LogMAR) pre and final post-trauma was 0.6 (SD 0.9; range − 0.1–2.7) and 1.7 (SD 1.0; range 0–2.7) respectively (*P* = 0.001). Furthermore, there was a threefold increase in number of patients with BCDVA worse than 6/60 (5 patients pre-trauma and 15 patients post-trauma) despite best management. The poor visual prognosis post-trauma in this cohort is illustrated in Fig. 1. The mean BCDVA at the pre-trauma stage in the non-trauma eye was 0.4 (SD 0.5; range − 0.1–2.7). The difference in BCDVA between the two eyes was not significantly different (*P* > 0.05). The final BCDVA was 6/9 or better in 4 eyes (19%) that experienced trauma.

#### Eye trauma

The episodes of globe rupture occurred equally in the right eye and left eye, each experiencing 11 episodes. Globe rupture occurred at the graft–host junction in all patients but one. In this case, a post-trauma full thickness rupture occurred in an area of pre-existing corneal thinning due to a previous melt. Data for one patient at the time of trauma was missing. Graft–host junction dehiscence occurred in one quadrant (*n* = 5), in two quadrants (*n* = 13), in three quadrants (*n* = 2) and there were no instances of four quadrant dehiscence. The specific location of the graft–host junction dehiscence was in the quadrants superior–temporal (*n* = 8), superior-nasal (*n* = 7), inferior-temporal (*n* = 10) and inferior-nasal (*n* = 12). There was no statistical difference between the inferior and superior location of the graft-host dehiscence, *P* = 0.25.

#### Graft status

As a result of the globe rupture, de novo graft failure was noted in 11 grafts (60.9%) following trauma, at < 1 year post-trauma in 9 patients (81.8%) and at > 1 year post-trauma in 2 (18.2%) patients. The post globe rupture probability of corneal graft survival was 0.52, 0.42 and 0.33 at 1, 5, and 10 years, respectively. Repeat PK was performed on 5 out of 11 (45.5%) of these failed grafts. Amongst these five patients, two patients had a final BCDVA of ≥ 6/12 (0.3 LogMAR), and three patients resulted in graft failure. One of these three patients had a visual outcome of hand movements due to subsequent endothelial failure and chronic glaucoma. The other two patients suffered an episode of graft rejection with one patient having a BCDVA of 6/60 (1.0 LogMAR), and the other patient having a visual outcome of hand movements. Various factors were evaluated to analyse their effect on graft survival after globe rupture and there was no statistical significance (*P* ≥ 0.05) when comparing sex, age, the original indication for grafting, or the time interval between grafting and trauma.

#### Post-trauma associated ocular complications

All patients but one underwent primary globe repair emergency surgery and management of the concomitant anterior and posterior segment trauma as deemed necessary. One patient underwent primary evisceration due to extensive damage to the eye with severe extrusion of intraocular structures and visual acuity of no perception of light at the time of presentation. In the study data analysis, this patient was included in the failed graft cohort. All primary repairs but one were undertaken within 24 h from the trauma. One patient underwent surgical repair at 8 days post-trauma due to a delay in presentation. Ocular injury with lens damage (both phakic and pseudophakic) was associated with significantly poorer final BCDVA than eyes with no lens damage (*P* < 0.05). A summary of primary (occurring at time of trauma) and secondary (occurring at the follow-up stage) ocular complications resulting from the trauma are described in Table 5.

**Table 5:** Primary and secondary ocular complications

Primary Complications	*n* (%)
*Anterior Segment*	
Lens	
Phakic subluxation	1 (4.5)
PCIOL expulsion	6 (27.3)
Phakic lens expulsion	6 (27.3)
Iris	
Prolapse	8 (36.4)
Damage	2 (9.1)
Dialysis	1 (4.5)
*Posterior Segment*	
Vitreous	
Haemorrhage	3 (13.6)
Prolapse into AC	2 (9.1)
Prolapse into wound*	7 (31.8)
Choroidal detachment	2 (9.1)
Retinal detachment^α^	2 (9.1)

Secondary complications	*n* (%)
Aphakia	11 (50.0)
Glaucoma	8 (36.4)
Total retinal detachment	7 (31.8)
Hypotony	5 (22.7)
Wound leak	1 (4.5)
Cystoid macular oedema	1 (4.5)
Macular scarring	1 (4.5)
Preseptal cellulitis	1 (4.5)
Endophthalmitis	1 (4.5)

*Phakic (n = 5), Aphakia (n = 1), Pseudophakia (n = 1)

^α^Not repaired at time of primary repair

## Discussion

Over the last 10 years this is the first published series of traumatic globe rupture after corneal transplantation from a large tertiary referral centre in the UK. Previously reported large case series from the UK were from Glasgow [10] and Belfast [11], reporting a 6-year incidence of 3.8%, and a 12-year incidence of 2.6%, respectively. Our incidence of traumatic globe rupture was 3.0%, which is in keeping with the published literature which reports an incidence range from 1.4 [12] to 5.8% [13]. Our incidence may even be higher due to the possibility that a few patients were lost to follow-up including four deceased patients, two patients who moved out of the area and one that failed to attend follow-up appointments.

The demographic age in our case series compared to that from Glasgow and Belfast is similar, however the demographic gender is dissimilar. Our series includes 12 males and 9 females, the Belfast study reports a higher number of females 8, and 7 males, and the Glasgow study reports 14 males and 4 females. Epidemiology studies have shown the incidence of ocular trauma in males is higher than in females, ranging from 2 to 5.5 times higher. The higher incidence is thought to be related to occupational hazards and risk-taking behaviour amongst males [14–16].

The latency from surgery to the episode of trauma ranged from as early as 3 months to 9 years, indicating that a risk of wound dehiscence after trauma is possible during the entire lifetime of the graft [13, 17]. Clinical and experimental studies suggest that scar tissue at the graft–host junction never regains the full strength of normal corneal tissue and consequently traumatic globe rupture remains a risk for the remainder of the patient's life [4, 18]. However, the risk of globe rupture is highest in the period immediately following surgery, when wound strength is derived almost entirely from sutures, and at later stages during the period immediately following removal of sutures [13]. Numerous factors have been proposed to be associated with a weak host-graft junction in PK patients, which predisposes to traumatic wound dehiscence. These include corneal avascularity and prolonged treatment with topical steroids, leading to less wound scarring and delayed wound healing, respectively [19, 20]. However, there has been reports showing that graft-host junction structural integrity remains weak even with associated vascularization [21]. Simonsen et al. reported that following penetrating keratoplasty, the tensile strength of the corneal wound is 50% that of the normal intact tissue 2 to 3 years postoperatively [22]. The corneal wound edges are reconnected by new collagen fibrils deposited within the wound that intercalate the adjacent stromal lamellae rather than by reanastomosis [23]. Graft dehiscence occurred more commonly but not significantly in the inferior quadrants in our series. Published reports are contradictory, some indicate no specific quadrant of the globe is more predisposed to rupture [24, 25] whilst others postulate that because the globe is least protected inferiorly by the bony orbit, blunt trauma in turn may transfer the pressure to the opposite site (superior) of the cornea, resulting in more frequent superior graft dehiscence [26]. All the patients that experienced globe rupture in our series had some remaining corneal sutures including interrupted, continuous and some patients even had a combination of continuous and interrupted sutures still in place. As per our protocol, only corneal sutures that loosen, break or induce astigmatism are routinely removed. Hence interpretation of the impact is unclear. From the literature the impact of sutures is controversial; Kawashima et al. reported in their large study (*n* = 43) no significant relationship between the presence or absence of sutures and wound dehiscence [17]. However, other authors have reported a high-risk period for graft wound dehiscence the month following removal of sutures [13].

All patients underwent primary repair within 24 h of presentation to the eye emergency service with the exception of one who underwent repair 8 days post-trauma. This patient was originally admitted under the care of the medical team having suffered a fall that required emergency general medical care. A diagnosis of traumatic globe rupture and aphakia (IOL expulsion) was only made 8 days post-trauma when a computerized tomography (CT) scan of the head was undertaken to exclude significant head injury. The radiology report suggested the possibility of unilateral globe rupture that instigated an urgent referral to ophthalmology for an emergency assessment. The BCDVA for this patient 6 months following globe repair was perception of light due to graft failure.

The indication for original grafting did not influence the risk of future traumatic globe rupture, although the numbers were relatively small for each separate original indication for grafting. Other series have also reported no association [13]. The most frequent cause of blunt ocular trauma was as a result of falling, and the mean age of those falling was significantly higher compared to other mechanisms of injury, this has been noted in other studies [17]. However, the cause of ocular injury amongst younger individual was more commonly as a result of accidental blunt trauma or assault, similar to other studies [11, 27. Our study shows a bimodal relationship with age and cause of traumatic globe rupture post-corneal graft. The mean age of those falling was 71.5 years (SD 14.8), which was significantly higher when compared to the other mechanisms of trauma, with mean age being 45.3 years (SD 20.8), *P* < 0.05. Williams and colleagues [11] pooled data from previously published case series, as their case numbers were small (*n* = 15) and were first to identify a bimodal relationship between age and mechanism of trauma in patients having a previous keratoplasty. They reported the mechanism of trauma in patients > 50 years was most often as a result of falls, and those < 50 years most often as a result of assault and accidental sport-related injuries. A disadvantage of the pooled previously published case series method is accepting incomplete or unclear published data, e.g. in some cases the exact nature of the accidental trauma was unclear, trauma was simply described as a domestic accident, which could include falls. We have one missing data on mechanism of trauma, and therefore our findings offer stronger evidence of the bimodal relationship between mechanism of injury and age.

This case series demonstrates that the visual prognosis following traumatic globe rupture after keratoplasty is rather poor with 15/21 (71.4%) of patients having a final BCDVA of 6/60 or worse. Similarly, poor visual prognoses have been previously reported in similar studies, e.g. 73% (*n* = 26 grafts) of visual outcomes had a BCDVA ≤ 6/60, [28] and 65% (n = 36 grafts) with VA of < 6/60 [17] The poor BCDVA in most patients was as a result of graft failure and secondary complications, such as posterior segment involvement and glaucoma. The association between a poor final visual acuity and posterior segment involvement has been previously reported [13, 17, 25, 27].

We noted that the risk of losing the intraocular lens in phakic and pseudophakic patients was of 31.8% and 27.3%, respectively. The associated risk of graft failure in phakia and pseudophakic patients was of 42.9% and 50.0%, respectively, even if the lens was not directly involved in the trauma. Our results are clinically important, as we have shown that ruptured globes with lens damage are associated with final poorer visual acuity as a result of associated posterior segment complications and graft failure [17]. Lens damage may therefore serve as a clinical prognostic indicator of poor final visual outcome.

One patient underwent primary evisceration due to the severe extent of the ocular trauma with intraocular tissue loss and no perception of light on presentation. Fortunately this is a very rare outcome, with a reported small number of patients 0.17% (*n* = 3) undergoing primary evisceration post PK globe rupture [29].

Graft failure occurred most commonly within the first 3 months post-trauma, and this is likely to be as a result of direct traumatic damage to the corneal endothelium. Only one patient suffered a rejection episode at 1-year post-trauma, which resolved successfully but graft failure happened at 2 years post-trauma. Re-grafts were performed in five patients. The final BCDVA of ≥ 6/12 was achieved in 40% of these patients. Re-grafts were performed in only patients with no or minor posterior segment complications and may explain the good visual outcomes. Our post globe rupture probability of corneal graft survival was 0.52, 0.42 and 0.33 at 1, 5, and 10 years, respectively. The Australian Corneal Graft Registry (ACGR) is a well-published large case series that reports the probability of corneal graft survival as 0.87, 0.73 and 0.60 at 1, 5 and 10 years, respectively, in all causes of graft failure. The ACGR do not specifically report outcomes in trauma specific causes. We would expect in trauma cases, the corneal graft survival to be worse, and is indeed lower in our study [30].

In our series, no patients experienced globe rupture following previous DSAEK or DALK surgery. This may be in part due to the relatively small numbers of these types of lamellar keratoplasties in our case series. Furthermore, the trend towards lamellar keratoplasties may provide a higher level of mechanical globe protection [31, 32]. As previously highlighted one of the limitations of this study includes the possibility of under-reporting, because of the odd chance that patients may have moved out of the region or completely lost to follow-up.

## Conclusions

In summary, this 20-years review study shows that globe rupture following PK is fortunately a rather uncommon but serious eye complication that often results in poor visual outcomes and graft failure in most patients. At presentation, urgent primary repair of the ruptured globe and proper counselling regarding prognosis are essential. BCDVA, lens status and the extent of posterior segment involvement at presentation must be factored in when counselling the patient before emergency surgical intervention. Re-grafting in select patients with good prognostic factors, such as an intact lens status and no posterior segment complications, may offer the patient valuable visual rehabilitation. Counselling patients regularly about their potential serious risk of globe rupture after trauma particularly in PK patients and addressing strategies to reduce this risk are essential. These include recommending the use of protective eyewear, assessing contributing risk factors, such as the patients’ age, general health and associated falls risk. It is rather important based on our data to pay special attention to elder-friendly and safe home setup, including well-lighted rooms, safe stairs, friendly floors and home support as required. A heightened awareness amongst general medical colleagues of the risk of globe rupture in case of falls in the older patient group after PK is warranted, and a very low threshold for suspecting ruptured globe after trauma and requesting urgent ophthalmic assessment in these patients is highly recommended.
